# A surface renewal model for unsteady-state mass transfer using the generalized Danckwerts age distribution function

**DOI:** 10.1098/rsos.172423

**Published:** 2018-05-23

**Authors:** Isabelle R. Horvath, Siddharth G. Chatterjee

**Affiliations:** 1 Department of Environmental Resources Engineering, SUNY College of Environmental Science and Forestry, 1 Forestry Drive, Syracuse, NY, USA; 2 Department of Paper and Bioprocess Engineering, SUNY College of Environmental Science and Forestry, 1 Forestry Drive, Syracuse, NY, USA

**Keywords:** Danckwerts distribution, mass transfer, surface renewal model, turbulence

## Abstract

The recently derived steady-state generalized Danckwerts age distribution is extended to unsteady-state conditions. For three different wind speeds used by researchers on air–water heat exchange on the Heidelberg Aeolotron, calculations reveal that the distribution has a sharp peak during the initial moments, but flattens out and acquires a bell-shaped character with process time, with the time taken to attain a steady-state profile being a strong and inverse function of wind speed. With increasing wind speed, the age distribution narrows significantly, its skewness decreases and its peak becomes larger. The mean eddy renewal time increases linearly with process time initially but approaches a final steady-state value asymptotically, which decreases dramatically with increased wind speed. Using the distribution to analyse the transient absorption of a gas into a large body of liquid, assuming negligible gas-side mass-transfer resistance, estimates are made of the gas-absorption and dissolved-gas transfer coefficients for oxygen absorption in water at 25°C for the three different wind speeds. Under unsteady-state conditions, these two coefficients show an inverse behaviour, indicating a heightened accumulation of dissolved gas in the surface elements, especially during the initial moments of absorption. However, the two mass-transfer coefficients start merging together as the steady state is approached. Theoretical predictions of the steady-state mass-transfer coefficient or transfer velocity are in fair agreement (average absolute error of prediction = 18.1%) with some experimental measurements of the same for the nitrous oxide–water system at 20°C that were made in the Heidelberg Aeolotron.

## 1. Introduction

The surface of a turbulent liquid is characterized by bursting and chaotic movements of eddies that come from beneath the surface, and by the presence of turbulent sweeps, upwellings, downwellings and vortices that profoundly influence the interfacial mass-transfer process [1–3]. In 1951, Danckwerts [4] presented his classic surface renewal model of mass transfer to quantitatively describe gas absorption at the surface of a turbulent liquid. This model visualizes the gas–liquid interface, where the absorption occurs, to be continuously rejuvenated by fresh liquid elements arriving from the bulk liquid. Danckwerts derived an exponential age distribution by using the postulate that all surface elements, irrespective of their individual ages, had equal probability of being replaced by fresh elements arriving at the surface from the bulk liquid. The only parameter of this distribution is the frequency or rate of surface renewal *S*, which Danckwerts called the mean rate of production of fresh surface, and which depends upon the prevailing hydrodynamic conditions. This well-known age distribution, whose experimental confirmation has been provided by Lamb *et al.* [5] for the case of a stirred liquid and by Lesage *et al.* [6] in the case of pipe flow, has been widely used in chemical engineering, and a variety of applications and extensions of the surface renewal model has appeared in the literature over the years [7–18]. According to Komori *et al.* [1], large-scale, surface renewal eddies dominate mass transfer across a gas–liquid interface, with the liquid-side mass-transfer coefficient being proportional to the square root of the surface renewal frequency. Also, according to Banerjee [2], liquid-side controlled gas exchange for clean, non-breaking interfaces is well predicted by surface renewal models with the renewal frequency being that of the turbulent sweeps that impinge on the interface.

In many mass-transfer studies it has been reported that the (steady-state) liquid-side mass-transfer coefficient or transfer velocity $k_{L}\propto Sc^{-m}$ (i.e. $k_{L}\propto D^{m})$,where *Sc* is the Schmidt number, which is equal to *ν/D*, with *ν* and *D* being the kinematic viscosity of the liquid and the diffusion coefficient of the dissolved gas or solute in the liquid, respectively (table 1). The value of *m* generally lies in the range of 0.5 (penetration and surface renewal models) to 1 (film model). According to Astarita [19], in the case of a liquid in contact with a solid or a more viscous liquid phase, $k_{L}\propto D^{2/3}$. Kuthan & Brož [20] obtained experimental values of *k*_L_ for the absorption of helium, nitrogen and propane by a liquid film of aqueous ethylene glycol flowing over a smooth wetted wall and an expanded metal sheet. For the case of the wetted wall, they found $k_{L}\propto D^{0.5}$, which is in agreement with the surface renewal model. For the expanded metal sheet, $k_{L}\propto D^{0.64}$ and the film-penetration model, proposed in 1958 by Toor & Marchello [21], which uses the conventional (i.e. exponential) Danckwerts age distribution function, was found to be more appropriate. Richter & Jähne [22] measured the transfer velocity (i.e. *k*_L_) of five sparingly soluble gas tracers as a function of wind speed (1–10 m s^−1^) in the Heidelberg Aeolotron and in a small circular wind-wave facility. Their experiments showed that the Schmidt number exponent (i.e. −*m*) varied from −2/3 to −1/2 as the water surface transitioned from smooth to rough or wavy with increasing wind speed. The measured transfer velocity ranged from about 2.78 × 10^−6^ m s^−1^ (1 cm h^−1^) to 1.94 × 10^−4^ m s^−1^ (70 cm h^−1^), as estimated from fig. 4 in their paper. Krall [23] reported extensive measurements of the transfer velocity of five sparingly soluble gases in the Heidelberg Aeolotron and the Kyoto high-speed wind-wave tank at wind speeds between 1.2 and 67 m s^−1^, and confirmed the above dependency of the Schmidt number exponent. The measured transfer velocities spanned more than three orders of magnitude and lay between 1.39 × 10^−6^ m s^−1^ (0.5 cm h^−1^) and 3.06 × 10^−3^ m s^−1^ (1100 cm h^−1^).

**Table 1.** Nomenclature.

*a*	parameter of the generalized Danckwerts age distribution
*c*(*x, t*)	dissolved-gas concentration in a surface element at location *x* and time *t*, kmol m^−3^
*c*_b_	dissolved-gas concentration in the bulk liquid, kmol m^−3^
*c*_s_	dissolved-gas concentration at the gas–liquid interface, kmol m^−3^
*C*_1_–*C*_4_	constants in equations (1.1) and (1.2)
*D*	diffusion coefficient of the dissolved gas or solute in the liquid, m^2^ s^−1^
*E*	rate of energy dissipation per unit mass of fluid, W kg^−1^
*f* (*t, t*_p_)	age distribution of surface elements at process time *t*_p_, s^−1^
*k*_L_	steady-state liquid-side mass-transfer coefficient or transfer velocity, m s^−1^ or cm h^−1^
$k_{L}^{\mathrm{abs}}(t_{p})$	liquid-side gas-absorption coefficient, m s^−1^
$k_{L}^{\mathrm{trans}}(t_{p})$	liquid-side dissolved-gas transfer coefficient, m s^−1^
*K*	given by equation (2.4)
*L*	average thickness of a surface element, m
*m*	negative of the Schmidt number exponent
*M*_s_	modulus of surface elasticity of the liquid or fluid, kg s^−2^
*R*_abs_(*t*_p_)	average rate of gas absorption at process time *t*_p_, kmol m^−2^ s^−1^
*R*_inst_(*t*)	instantaneous rate of gas absorption in a surface element having an age of *t*, kmol m^−2^ s^−1^
*R*_trans_(*t*_p_)	average rate of transfer of dissolved gas to the bulk liquid at process time *t*_p_, kmol m^−2^ s^−1^
*S*	fundamental renewal frequency of surface elements, s^−1^
*Sc*	Schmidt number (*ν*/*D*)
*t*	age of a surface element, s
*t*_p_	process time, s
*t*_ren_	mean eddy renewal or burst time, s
*x*	distance into the liquid measured from the gas–liquid interface, m
*y*	parameter of *Γ*(*z*, *y*)
*z*	parameter of *Γ*(*z*, *y*)
*Greek letters*	
*α*	defined by equation (4.2)
*Γ*(*z*, *y*)	extended Euler gamma function (defined by equation (2.5))
*δ*(*t*)	delta function at *t* = 0
*λ*	variable of integration
*ν*	kinematic viscosity of liquid or fluid, m^2^ s^−1^
*ρ*	density of liquid or fluid, kg m^−3^

To explain the dependency of *k*_L_ on the Schmidt number exponent, we now provide a brief overview of the film-penetration model [21]. This model will also be alluded to later in relation to the surface renewal model presented in this work According to the film-penetration model, the film and penetration models represent two limiting descriptions of the absorption process. At low Schmidt numbers, a steady-state concentration gradient is set up very quickly in a surface element, and mass transfer occurs through what is essentially a film (i.e. *m* = 1), with surface renewal playing an insignificant role. As the Schmidt number increases, the time required to establish a steady-state concentration gradient in the element increases, with most of the surface elements not being penetrated completely by the dissolved gas. The mass transfer then follows the penetration or surface renewal model (i.e. *m* = 0.5). When the surface contains significant amounts of young, middle-aged and old elements, the mass-transfer characteristics are intermediate between the film and penetration models (i.e. 0.5 < *m* < 1). Using the film-penetration model, Toor & Marchello [21] analysed the case of mass transfer from a solid to a fluid in turbulent flow and showed that, for *Sc* less than 10, $k_{L}\propto D$, while for *Sc* greater than about 1000, $k_{L}\propto D^{0.5}$ with *m* gradually changing from 1 to 0.5 in the range 100<Sc<1000. This behaviour was consistent with mass-transfer rate data for flow in conduits over the full range of Schmidt numbers. The film-penetration model contains two parameters. These are the rate of surface renewal of liquid elements (*S*) and the characteristic size of a liquid element (*L*), both of which are assumed to depend on the prevailing hydrodynamic conditions. In a detailed study, Metzger & Dobbins [24] presented the following theoretical equations for the film-penetration model:

1.1 $$\begin{array}{rl} S & =\frac{C_{1}{C_{2}}^{3/4}{C_{3}}^{3}\rho \nu^{3/4}E^{3/4}}{M_{s}}, \end{array}$$

1.2 $$\begin{array}{rl} L & =C_{4}{\left(\frac{\nu^{3}}{E}\right)}^{1/4} \end{array}$$

1.3 $$\begin{array}{rl} \text{and}\;k_{L} & =\sqrt{DS}\coth\left(\sqrt{\frac{SL^{2}}{D}}\right). \end{array}$$

where *C*_1_–*C*_4_ are constants, *ρ* is the density of the fluid, *ν*, as mentioned earlier, is the kinematic viscosity of the fluid, *M*_s_ is the modulus of surface elasticity of the fluid, *L* is the average thickness or size of a surface element and *E* is the specific rate of energy dissipation in the fluid as a whole (due to turbulent mixing). Metzger & Dobbins [24] derived equation (1.1) by equating the resisting pressure (which opposes eddy motion) at the surface of the fluid to the product of the fluid density and the square of the eddy velocity, and by assuming that this velocity is proportional to the Kolomogoroff velocity factor (which is equal to the product of the kinematic viscosity of the fluid and the energy dissipation rate per unit mass of fluid raised to a power of 0.25). Metzger & Dobbins [24] also verified equations (1.1) and (1.2) by performing experiments on the absorption of helium, nitrogen and oxygen in water contained in an agitated absorption cylinder. For the absorption of oxygen in water (i.e. aeration), they found that the film-penetration model was able to correctly account for the influence of temperature on the absorption coefficient. According to equation (1.3), as the dimensionless group *SL*^2^/*D *→ 0, *k*_L _→ *D*/*L* (film model; *m* = 1), while as it → ∞ (larger than 3 practically), *k*_L _→ $\sqrt{DS}$ (surface renewal model; *m* = 0.5). Brusset *et al.* [25] developed correlations for *S* and *L* from prior experimental data on the evaporation of liquids into a turbulent gas stream in a wetted-wall column. These correlations, for which they provided theoretical justification, show power law-type dependency of the parameters on the gas-phase Reynolds number. The film-penetration model was able to explain the empirical observation $k_{L}\propto D^{m}$ where, as mentioned earlier, *m* lies in the range 0.5 to 1. Chatterjee & Altwicker [26] re-examined the data of Brusset *et al.* [25] and showed that *SL*^2^/*D* varied linearly with the gas-phase Reynolds number. Briens *et al.* [27] experimentally measured particle–liquid heat- and mass-transfer coefficients in packed and fluidized beds of glass beads and obtained values of *S* and *L* as functions of liquid superficial velocity, gas velocity and radial location in the bed. For packed and fluidized beds of particles, they found that the Chilton–Colburn analogy (which relates the heat- and mass-transfer coefficients) was not adequate and recommended the film-penetration model for accurate predictions. Krishna [28] generalized the film-penetration model to multicomponent mass transfer and analysed the non-ideal acetone–benzene–methanol system. The film model predicted a constant negative flux of benzene (i.e. transfer of benzene in a direction opposite to that of its imposed concentration gradient), whereas the penetration model predicted a positive value for this flux, which decreased with time. The film-penetration model provided a smooth transition between the penetration and film models.

Recently, Mondal & Chatterjee [29] have given a deeper theoretical understanding of the Danckwerts surface renewal model by invoking the concept of a frequency quantum, which is analogous to the energy quantum postulate which Planck used to derive his famous formula for the energy density of black-body radiation. By assuming that surface elements of a particular age have renewal frequencies that are integral multiples of this fundamental frequency quantum, and by further assuming a Boltzmann-type distribution for the renewal frequency, they showed that a population balance for these elements led to the Danckwerts surface age distribution. The basic quantum is what has been conventionally called the rate of surface renewal, *S*. The Higbie surface age distribution resulted if the renewal frequency distribution of such elements was assumed to be continuous. They also derived four different unsteady-state age distributions, which corresponded to four different hypotheses about the behaviour of liquid elements at the gas–liquid interface, and which reflected different initial states of the interface when the absorption (i.e. mass transfer) commenced. The first two age distributions were two different versions of the traditional Danckwerts model, the third one was based on the uniform and Higbie distributions, whereas the fourth was a mixed distribution. For all four cases they derived explicit mathematical expressions for the rates of physical gas absorption at the gas–liquid interface and dissolved-gas transfer to the bulk liquid from the surface. Mondal & Chatterjee [29] showed that, under unsteady-state conditions, these two rates were not equal but had an inverse relationship, and only with the progress of absorption towards steady state did they approach one another.

Some studies by the physical oceanography community have reported that the age distribution of surface elements in air–sea heat and gas exchange, as measured both directly and indirectly, does not follow the (conventional) exponential age distribution of Danckwerts but rather the logarithmic normal (LN) or Chi distributions [30–32]. Assuming steady-state conditions, Mondal & Chatterjee [29] generalized the conventional one-parameter Danckwerts age distribution to a two-parameter age distribution, which they called the generalized Danckwerts (GD) age distribution. They achieved this by introducing a time lag into the basic differential equation that describes the conventional Danckwerts distribution. Like the two-parameter LN distribution, the GD distribution was also able to capture the bell-shaped nature of experimentally measured surface age distribution, which, as mentioned earlier, has been observed in air–sea gas and heat exchange. Mondal & Chatterjee [29] used the LN and GD distributions to calculate the steady-state liquid-side mass-transfer coefficient for the absorption of hydrogen and oxygen in water at three different wind speeds as used by Garbe *et al.* [30] in their experiments on air–water heat exchange on the Heidelberg Aeolotron. They found that, for both distributions, estimates of the mass-transfer coefficient were very close to one another and comparable to the experimental values reported by Hutchinson & Sherwood [33] who investigated the absorption of eight different pure gases at 25°C in a stirred flask containing water whose surface was exposed to the gas.

The chief objective of the present work is to extend the two-parameter GD age distribution beyond the steady-state form derived by Mondal & Chatterjee [29], to unsteady-state conditions. This distribution is then used to analyse the unsteady-state absorption of a gas into a large volume of liquid, assuming negligible gas-side mass-transfer resistance. Explicit mathematical expressions are derived for the unsteady-state liquid-side mass-transfer coefficients for the absorption of the gas at the gas–liquid interface and its subsequent transfer to the bulk liquid. These expressions are used to calculate the dynamic liquid-side gas-absorption and dissolved-gas transfer coefficients for the absorption of oxygen in water at 25°C for the three different experimental wind speeds used by Garbe *et al.* [30]. Finally, some comparisons are made between theoretical predictions of the steady-state mass-transfer coefficient (i.e. transfer velocity) and experimental measurements of the same that were made by Krall [23] in the Heidelberg Aeolotron using the nitrous oxide–water system at 20°C.

The foreseeable applications of the GD age distribution are in the modelling of gas absorption in a gas–liquid reactor or packed tower and modelling of air–sea gas and heat exchange under unsteady- and steady-state conditions.

## 2. Unsteady-state GD age distribution

Using equations (A.8) and (A.15) in the appendix (i.e. electronic supplementary material, S1) of the manuscript of Mondal & Chatterjee [29] and generalizing them to unsteady-state conditions yields the fundamental differential equation that describes the unsteady-state GD age distribution function *f*(*t*, *t*_p_):

2.1 $$\frac{\partial f}{\partial t}+Sf\left(2a+1-\frac{a}{St}\right)=0,$$

where *t* is the age of a surface element and *t*_p_ is the time elapsed since the commencement of the mass or heat transfer process (i.e. the process or ‘clock’ time). The two parameters of the model are *S* (fundamental frequency quantum, alluded to earlier) and *a*, which is related to the width of the distribution as will be discussed later. The solution of this equation is given by

2.2 $$f(t,t_{p})=Kt^{a}{\text{e}}^{-(2a+1)St},$$

where *K* is a parameter. As

2.3 $$\int_{0}^{t_{p}}f(t,t_{p})\text{d}t=1,$$

substitution of equation (2.2) into equation (2.3) yields

2.4 $$K=\frac{{\left[(2a+1)S\right]}^{a+1}}{\Gamma (a+1)-\Gamma (a+1,(2a+1)St_{p})},$$

where *Γ*(*z*, *y*) is the extended Euler gamma function defined by

2.5 $$\Gamma \text{(}z,y\text{)}=\int_{y}^{\infty }\lambda^{z-1}{\text{e}}^{-\lambda }\text{d}\lambda .$$

Using equation (2.4) in equation (2.2) yields the unsteady-state GD age distribution function, i.e.

2.6 $$f(t,t_{p})=\frac{S{\left(2a+1\right)}^{a+1}{\left(St\right)}^{a}{\text{e}}^{-(2a+1)St}}{\Gamma \text{(}a+1\text{)}-\Gamma (a+1,(2a+1)St_{p})}.$$

Letting *a* = 0 and using the relations Γ(1)=1 and $\Gamma (1,St_{p})={\text{e}}^{-St_{p}}$ reduces equation (2.6) to

2.6a $$f(t,t_{p})=\frac{Se^{-St}}{1-{\text{e}}^{-St_{p}}},$$

which is the (exponential) unsteady-state Danckwerts age distribution for Case 1 that was previously derived by Mondal & Chatterjee [29]—see equation (2.11) in their paper. For this case, as was mentioned by these authors, the gas–liquid interface is assumed to be instantaneously and completely formed at *t*_p_ = 0 with liquid elements flowing into it from the bulk liquid and departing from it to the bulk liquid at a constant rate for *t*_p_ ≥ 0.

As $t_{p}\to \infty$, equation (2.6) simplifies to

2.7 $$f(t,t_{p}\to \infty )=S\frac{{\left(2a+1\right)}^{a+1}}{\Gamma \text{(}a+1\text{)}}{\left(St\right)}^{a}{\text{e}}^{-(2a+1)St},$$

which is the steady-state GD age distribution derived before by Mondal & Chatterjee [29]. For *a* = 0, equation (2.7) reduces to $f(t,t_{p}\to \infty )=Se^{-St}$, which is the conventional (i.e. exponential) Danckwerts age distribution.

The mean eddy renewal or burst time *t*_ren_ can be obtained from

2.8 $$t_{\mathrm{ren}}(t_{p})=\int_{0}^{t_{p}}tf(t,t_{p}\text{) d}t.$$

By substituting equation (2.6) into equation (2.8), it can be shown that

2.9 $$t_{\mathrm{ren}}(t_{p})=\frac{1}{S(2a+1)}\left[\frac{\Gamma \text{(}a+2\text{)}-\Gamma (a+2,(2a+1)St_{p})}{\Gamma (a+1)-\Gamma (a+1,(2a+1)St_{p})}\right].$$

For $t_{p}\to 0$, equation (2.9) becomes

2.10 $$t_{\mathrm{ren}}(t_{p}\to 0)=\left(\frac{a+1}{a+2}\right)t_{p},$$

i.e. during the initial moments, the mean eddy renewal time is independent of the fundamental frequency quantum *S* and increases linearly with process time *t*_p_. For *a* = 0, equation (2.10) predicts that $t_{\mathrm{ren}}(t_{p}\to 0)=0.5t_{p}$. As $t_{p}\to \infty$, i.e. as the steady state is approached, equation (2.9) simplifies to

2.11 $$t_{\mathrm{ren}}(t_{p}\to \infty )=\frac{1}{S}\left(\frac{a+1}{2a+1}\right),$$

which result has been previously shown by Mondal & Chatterjee [29]. For *a* = 0 (i.e. the conventional Danckwerts age distribution), equation (2.11) gives $t_{\mathrm{ren}}(t_{p}\to \infty )=1/S$, i.e. the average eddy renewal time *t*_ren_ (at steady state) is equal to the reciprocal of the fundamental frequency quantum *S*, which is a well-known result. However, this is not true for other values of *a*.

## 3. Unsteady-state gas absorption in a large volume of liquid

Consider the physical transient absorption of a gas into a large body of liquid (i.e. constant bulk liquid concentration of dissolved gas). If the gas-side mass-transfer resistance is negligible, the concentration profile and instantaneous rate of absorption [*R*_inst_(*t*)] of the gas per unit area in a surface element (of infinite depth) having an age of *t* are given by [19,34]

3.1 $$\frac{c(x,t)-c_{b}}{c_{s}-c_{b}}=\text{erfc}\left(\frac{x}{2\sqrt{Dt}}\right)$$

and

3.2 $$R_{\mathrm{inst}}(t)=(c_{s}-c_{b})\sqrt{\frac{D}{\pi t}},$$

where *c*(*x, t*) is the dissolved-gas concentration in the element at location *x* (measured from the gas–liquid interface) and time *t*, *c*_s_ and *c*_b_ are the dissolved-gas surface and bulk liquid concentrations (assumed to be constants), respectively, and *D* is the diffusion coefficient of the dissolved gas in the liquid. The average rate of absorption [*R*_abs_(*t*_p_)] of the gas at process time *t*_p_ can be obtained from

3.3 $$R_{\mathrm{abs}}(t_{p})=\int_{0}^{t_{p}}R_{\mathrm{inst}}(t)f(t,t_{p})\;\text{d}t.$$

The transient net rate of transfer [*R*_trans_(*t*_p_)] of the dissolved gas to the bulk liquid for surface elements of infinite depth is given by [29]

3.4 $$R_{\mathrm{trans}}(t_{p})=S\int_{0}^{t_{p}}\int_{0}^{\infty }[c(x,t)-c_{b}]\;f(t,t_{p})\text{d}x\text{d}t,$$

where the first and second terms on the right-hand side of equation (3.4) represent the two opposite directions of convective mass transfer due to the surface renewal mechanism. These being first the convective transfer of dissolved gas to the bulk liquid from the gas–liquid interface, and second, that from the bulk liquid to the interface.

Upon substitution of equations (2.6) and (3.2) into equation (3.3), it can be shown that

3.5 $$R_{\mathrm{abs}}(t_{p})=k_{L}^{\mathrm{abs}}(t_{p})[c_{s}-c_{b}],$$

where $k_{L}^{\mathrm{abs}}(t_{p})$ is the dynamic liquid-side gas-absorption coefficient, which is given by

3.6 $$k_{L}^{\mathrm{abs}}(t_{p})=\sqrt{\frac{DS\text{(}2a+1\text{)}}{\pi }}\left[\frac{\Gamma \text{(}a+1/2\text{)}-\Gamma (a+1/2,(2a+1)St_{p})}{\Gamma (a+1)-\Gamma (a+1,(2a+1)St_{p})}\right].$$

Substituting *a* = 0 and using the relations $\Gamma (1/2)=\sqrt{\pi }$ and $\Gamma \text{(}1/2,St_{p}\text{)}=\sqrt{\pi }\left[1-\text{erf}\left(\sqrt{St_{p}}\right)\right]$ reduces equation (3.6) to

3.6a $$k_{L}^{\mathrm{abs}}(t_{p})=\sqrt{DS}\frac{\text{erf}(\sqrt{St_{p}})}{1-{\text{e}}^{-St_{p}}},$$

which has been derived previously by Mondal & Chatterjee [29] (see equations (3.6), (3.7) and (3.12) for Case 1 in their work). As $t_{p}\to 0$, equation (3.6) becomes

3.7 $$k_{L}^{\mathrm{abs}}(t_{p}\to 0)=2\left(\frac{a+1}{2a+1}\right)\sqrt{\frac{D}{\pi t_{p}}},$$

demonstrating that during the initial moments of absorption, the gas-absorption coefficient $k_{L}^{\mathrm{abs}}$ is independent of the surface renewal rate *S*.

Similarly, by substituting equations (2.6) and (3.1) into equation (3.4), and using the result

3.8 $$\int_{0}^{\infty }\text{erfc}\left(\frac{x}{2\sqrt{Dt}}\right)\text{d}x=2\sqrt{\frac{Dt}{\pi }},$$

it may be shown that

3.9 $$R_{\mathrm{trans}}(t_{p})=k_{L}^{\mathrm{trans}}(t_{p})[c_{s}-c_{b}],$$

where $k_{L}^{\mathrm{trans}}(t_{p})$ is the dynamic liquid-side dissolved-gas transfer coefficient, which is given by

3.10 $$k_{L}^{\mathrm{trans}}(t_{p})=2\sqrt{\frac{DS}{\pi (2a+1)}}\left[\frac{\Gamma (a+3/2)-\Gamma (a+3/2,(2a+1)St_{p})}{\Gamma (a+1)-\Gamma (a+1,(2a+1)St_{p})}\right].$$

Comparing equations (3.6) and (3.10) shows that their mathematical structures are different.

Substituting *a* = 0 and using the relation $\Gamma (3/2)=\sqrt{\pi }/2$ reduces equation (3.10) to

3.10a $$k_{L}^{\mathrm{trans}}(t_{p})=\sqrt{DS}\frac{1-(2/\sqrt{\pi })\Gamma (3/2,St_{p})}{1-{\text{e}}^{-St_{p}}},$$

which has been presented earlier by Mondal & Chatterjee [29] (see equations (3.6), (3.8) and (3.13) for Case 1 in their paper). For $t_{p}\to 0$, equation (3.10) reduces to

3.11 $$k_{L}^{\mathrm{trans}}(t_{p}\to 0)=4S\left(\frac{a+1}{2a+3}\right)\sqrt{\frac{Dt_{p}}{\pi }}$$

according to which, during the initial period of absorption, the dissolved-gas transfer coefficient, unlike the gas-absorption coefficient, is directly proportional to the surface renewal rate *S*.

As equations (3.7) and (3.11) indicate, for $t_{p}\to 0$, the gas-absorption coefficient $k_{L}^{\mathrm{abs}}$ is inversely proportional to $\sqrt{t_{p}}$, whereas the dissolved-gas transfer coefficient $k_{L}^{\mathrm{trans}}$ is directly proportional to it, i.e. they have an inverse relationship. A physical explanation for this behaviour, which is true in general, is forthcoming in this manuscript.

As $t_{p}\to \infty$, i.e. as the steady state is approached, equations (3.6) and (3.10) simplify to

3.12 $$k_{L}^{\mathrm{abs}}(t_{p}\to \infty )=k_{L}^{\mathrm{trans}}(t_{p}\to \infty )=k_{L}=\frac{\Gamma \text{(}a+1/2\text{)}}{\Gamma (a+1)}\sqrt{\frac{(2a+1)DS}{\pi }},$$

an expression which has also been presented before by Mondal & Chatterjee [29]. For *a* = 0 (i.e. the conventional Danckwerts model), equation (3.12) shows that (at steady state) the gas-absorption and dissolved-gas transfer coefficients coincide and become equal to $\sqrt{DS}$, which is a well-known result.

## 4. Results and discussion

As mentioned earlier, Garbe *et al*. [30] experimentally measured the age distribution of surface renewal events on the Heidelberg Aeolotron in air–water heat exchange. They found a good fit of the LN distribution to these data and reported values of the two parameters of this distribution at wind speeds of 2, 4.2 and 8 m s^−1^. Mondal & Chatterjee [29] showed that the two-parameter (steady-state) GD age distribution (equation (2.7)) was equivalent to the LN age distribution which Garbe *et al*. [30] had used to represent their experimental data, i.e. it could also capture the bell-shaped nature of the distribution of surface ages observed empirically by these researchers. The values of the parameters *a* and *S* of the GD distribution obtained by Mondal & Chatterjee [29] for the three different experimental wind speeds of Garbe *et al*. [30] are given in table 2. It is observed that both *a* and *S* increase with wind speed, i.e. with the turbulence intensity at the gas–liquid interface.

**Table 2.** Values of the parameters of the generalized Danckwerts age distribution function reported by Mondal & Chatterjee [29] for the experiments of Garbe *et al*. [30].

wind speed m s^−1^	*a*	*S* (s^−1^)
2.0	0.5	0.036
4.2	2.5	0.204
8.0	4.2	0.417

All calculations in this work were performed with the open-source mathematical software system SageMath—a sample code is provided in the electronic supplementary material.

**Figure 1.**
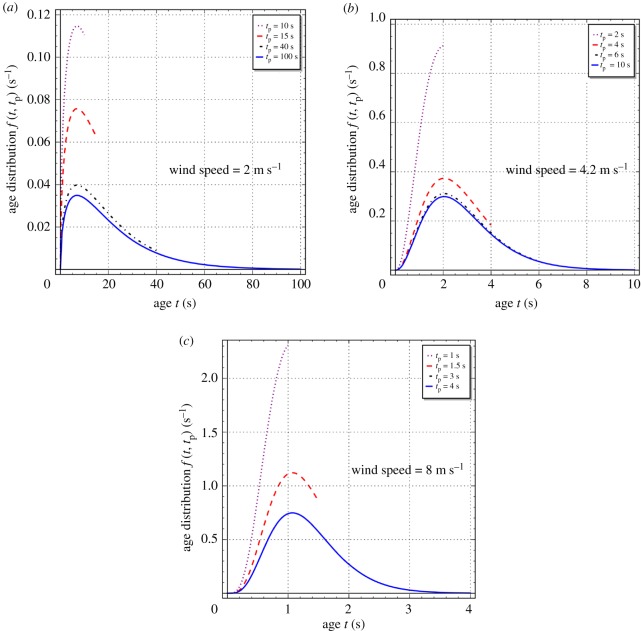
Unsteady-state generalized Danckwerts age distributions (equation (2.6)) for wind speeds of 2, 4.2 and 8 m s^−1^. Values of the parameters *a* and *S* are given in table 2.

Figure 1*a–c* shows unsteady-state GD age distributions calculated with equation (2.6) using the values of *a* and *S* reported in table 2 for wind speeds of 2, 4.2 and 8 m s^−1^. It can be clearly seen that, at each wind speed, the distribution has a sharp peak during the initial moments. However, with the progress of process time, it flattens out and acquires a bell-shaped character as the steady state is approached. The time taken to reach the steady state is a strong and inverse function of wind speed. It takes approximately 150, 9 and 4 s to reach the steady state at wind speeds of 2, 4.2 and 8 m s^−1^, respectively, which will be verified later. Thus, as the wind speed increases by a factor of 4, the time taken to reach the steady state decreases by a factor of 25. Further, as the wind speed increases, the age distribution narrows significantly, its skewness decreases and its peak becomes larger. This is because of an increase in the value of the parameter *a* with wind speed—see table 1 and fig. A1 in the appendix (electronic supplementary material, S1) of the manuscript of Mondal & Chatterjee [29]. It can therefore be postulated that the age distribution will acquire a delta function-like character at very high wind speeds (i.e. at high turbulence intensities).

**Figure 2.**
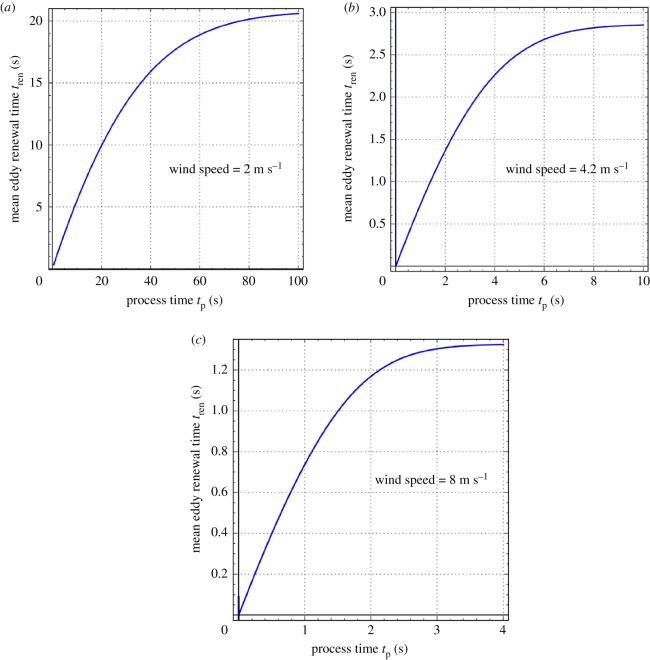
Behaviour of the mean eddy renewal or burst time with process time (equation (2.9)) for wind speeds of 2, 4.2 and 8 m s^−1^. Values of the parameters *a* and *S* are given in table 2.

In figure 2*a–c* the mean eddy renewal or burst time *t*_ren_ is plotted as a function of process time *t*_p_ (see equation (2.9)) at the three aforementioned wind speeds. As anticipated earlier from equation (2.10), *t*_ren_ increases in a linear fashion with *t*_p_ initially and then approaches a final steady-state value in an asymptotic manner. This steady-state value is practically 20.83, 2.86 and 1.33 s for the wind speeds of 2, 4.2 and 8 m s^−1^, respectively. Thus, a fourfold increase in wind speed decreases the steady-state value of *t*_ren_ by a factor of 16, which shows the great effect of turbulence on the hydrodynamics at the gas–liquid interface.

**Figure 3.**
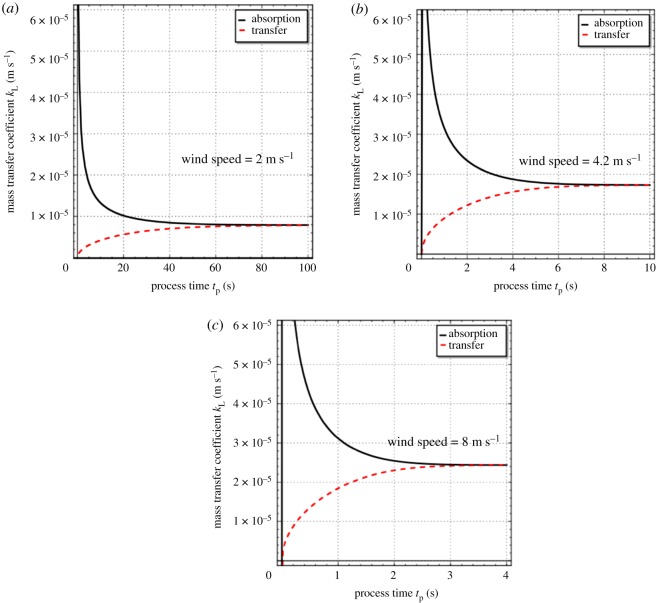
Behaviour of the gas-absorption coefficient (equation (3.6)) and dissolved-gas transfer coefficient (equation (3.10)) with process time for oxygen absorption in water at 25°C for wind speeds of 2, 4.2 and 8 m s^−1^. Values of the parameters *a* and *S* are given in table 2 and *D* = 2.12 × 10^−9^ m^2^ s^−1^.

Figure 3*a–c* exhibits the behaviour of the mass-transfer coefficients for gas absorption at the gas–liquid interface (equation (3.6)) and dissolved-gas transfer to the bulk liquid from the interface (equation (3.10)) as a function of process time *t*_p_ for the absorption of oxygen in water at 25°C for the three different wind speeds. At this temperature, the value of the diffusion coefficient *D* of oxygen in water is 2.12 × 10^−9^ m^2^ s^−1^ [29]. The gas-absorption coefficient shows an exponential decay-like behaviour with *t*_p_, decreasing from an initially high value to an asymptotic steady-state value as *t*_p_ increases. In sharp contrast, the dissolved-gas transfer coefficient increases smoothly from a value of 0 and merges with the gas-absorption coefficient curve as the steady state is approached (see equation (3.12)). To our knowledge, these two mass-transfer coefficients, which determine the rates of absorption at the gas–liquid interface and dissolved-gas transfer to the bulk liquid, have not been hitherto distinguished in the literature, the exception being the work of Mondal & Chatterjee [29].

The following explanation is advanced for the contrasting behaviour of the gas-absorption and dissolved-gas transfer coefficients with process time *t*_p_. During the initial moments of absorption, the gas–liquid interface will be populated with young elements, which will have a high absorption rate (see equation (3.2)). The gas-absorption coefficient $k_{L}^{\mathrm{abs}}$, which is an indication of the average rate of absorption according to equation (3.5), will therefore be high initially (see equations (2.6), (3.2) and (3.3)). However, young elements will also have a low dissolved-gas concentration (see equation (3.1)). The dissolved-gas transfer coefficient $k_{L}^{\mathrm{trans}}$, which is an indication of the average rate of dissolved-gas transfer to the bulk liquid according to equation (3.9), will hence be low during the initial moments of absorption (see equations (2.6), (3.1) and (3.4)). As the absorption process advances towards the steady state, the populations of young and old elements at the interface will more or less balance each other as the age distribution evolves with the passage of time and starts acquiring a bell-shaped character (figure 1*a–c*). The initially large divergence between these two coefficients will thus diminish as the steady state is approached. Mathematically, it is the dialectical interplay of equations (2.6), (3.1), (3.2), (3.3) and (3.4) which is responsible for this interesting behaviour.

The difference between $k_{L}^{\mathrm{abs}}$ and $k_{L}^{\mathrm{trans}}$ during the unsteady-state period of absorption implies a heightened accumulation of dissolved gas in the liquid elements at the surface of a turbulent liquid. This accumulation, which is expected to last from a few seconds to a few minutes depending upon the turbulence level, will be especially pronounced just after the start of the absorption process.

Table 3 reports the values of $k_{L}^{\mathrm{abs}}$ and $k_{L}^{\mathrm{trans}}$ for oxygen absorption in water at 25°C for the three different wind speeds. These values were obtained by performing calculations with equations (3.6), (3.10) and (3.12) using the sample SageMath code provided in the electronic supplementary material. The second column of this table shows the process time when both the gas-absorption and dissolved-gas transfer coefficients, calculated by equations (3.6) and (3.10), respectively, are almost equal to one another (and almost indistinguishable from the steady-state value calculated from equation (3.12) that is reported in the last column of the table), i.e. it is the time required to attain the steady state practically. It can be concluded from table 3 that a fourfold increase in wind speed increases the steady-state value of $k_{L}^{\mathrm{abs}}$ or $k_{L}^{\mathrm{trans}}$ by a factor of 3.1. The values in this table are also comparable to the values of 8.33 × 10^−6^ and 2.12 × 10^−5^ m s^−1^ for the liquid-side mass-transfer coefficient experimentally determined by Hutchinson & Sherwood [33] for the absorption of pure oxygen in a stirred flask containing water at 25°C (stirrer speed: 171 and 1025 r.p.m., respectively), with the stirred liquid surface being exposed to the gas.

**Table 3.** Calculated values of the gas-absorption and dissolved-gas transfer coefficients for oxygen absorption in water at 25°C. Values of the parameters *a* and *S* are given in table 2 and *D* = 2.12 × 10^−9^ m^2^ s^−1^.

wind speed m s^−1^	$t_{p}$ s	$k_{L}^{\mathrm{abs}}(t_{p})$ m s^−1^ (equation (3.6))	$k_{L}^{\mathrm{trans}}(t_{p})$ m s^−1^ (equation (3.10))	steady-state $k_{L}^{\mathrm{abs}}$ or $k_{L}^{\mathrm{trans}}$ m s^−1^ (equation (3.12))
2.0	150	7.87 × 10^−6^	7.86 × 10^−6^	7.87 × 10^−6^
4.2	9	1.73 × 10^−5^	1.73 × 10^−5^	1.73 × 10^−5^
8.0	4	2.44 × 10^−5^	2.43 × 10^−5^	2.44 × 10^−5^

**Figure 4.**
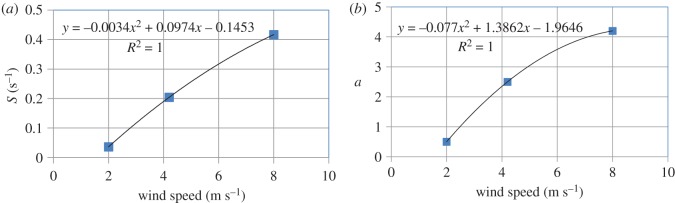
Variation of the parameters *S* and *a* of the generalized Danckwerts age distribution function with wind speed for the experiments of Garbe *et al*. [30]. Numerical values are shown in table 2.

Krall [23] conducted extensive theoretical and experimental investigations on the transfer velocities of a number of sparingly soluble gases in the Heidelberg Aeolotron and the Kyoto high-speed wind-wave tank. The fourth column of table 4 shows some of Krall's experimental data on transfer velocities for the nitrous oxide–water system at 20°C at different wind speeds. These data, which are for a clean water surface (i.e. no surfactant), were extracted from fig. 7.11 in Krall's work, which reports experimental transfer velocities (measured in the Aeolotron) as a function of the reference wind speed, which can be converted to the usually reported wind speed at a height of 10 m above the water surface by using fig. 7.9b in Krall's dissertation; these latter wind speeds are reported in the first column of table 4. The second and third columns of this table show values of the two parameters of the GD age distribution, *S* and *a*, at these wind speeds; these values were calculated from the correlations shown in figure 4*a*,*b*, which is a visual depiction of table 2. The diffusion coefficient of nitrous oxide in water at 25°C is 1.91 × 10^−9^ m^2^ s^−1^ [23]. Using the Wilke–Chang correlation [35] gives a value of *D* = 1.88 × 10^−9^ m^2^ s^−1^ for this system at the experimental temperature of 20°C used by Krall [23]. Values of the theoretical transfer velocity (*k*_L_) calculated from equation (3.12) are provided in the fifth column of table 4. The agreement between the theoretical and experimental values of the transfer velocity is quite reasonable (average absolute error of prediction = 18.1%) when the following facts are taken into consideration: (i) the values of the two parameters *S* and *a* were estimated from the experimentally measured age distribution of surface renewal events, obtained in the context of air–water heat exchange, on the Aeolotron that were reported by Garbe *et al.* [30] in 2002; (ii) the experimental data on transfer velocities were reported by Krall [23] in 2013, i.e. about a decade separates these two works during which period there was remodelling of the Aeolotron; and (iii) there is some uncertainty in the experimental measurements of the transfer velocity as can be seen in fig. 7.11 in the work of Krall [23]. Beyond a wind speed of 10 m s^−1^, the transfer velocity calculated from equation (3.12) was significantly lower than that measured experimentally. Two possible reasons for this behaviour are: (i) the correlations for *S* and *a* as functions of wind speed (figure 4*a*,*b*) are not valid because they only hold when the wind speed lies between 2 and 8 m s^−1^; and (ii) the air–water interface becomes broken at higher wind speeds with the appearance of bubbles and spray, and the surface renewal model, which is only applicable to a non-breaking surface, is no longer able to provide an adequate description of the ensuing complex mass transfer process.

**Table 4.** Experimental and theoretical values of the transfer velocity for the nitrous oxide–water system at 20°C. Values of the parameters *S* and *a* as functions of the wind speed were obtained as explained in the text and *D* = 1.88 × 10^−9^ m^2^ s^−1^. The experimental transfer velocities were extracted from fig. 7.11 in the work of Krall [23].

wind speed (at 10 m) m s^−1^	*S* s^−1^	*a*	experimental transfer velocity cm h^−1^	theoretical transfer velocity *k*_L_ (equation (3.12)) cm h^−1^	absolute error (%)
1.7	0.014	0.21	2.06	1.71	17.1
2.5	0.078	1.03	3.26	3.77	15.5
3.4	0.145	1.84	5.10	5.00	1.96
4.9	0.250	2.97	7.40	6.45	12.9
7.1	0.374	4.00	13.73	7.83	43.0
					average = 18.1%

As the theoretical values of the transfer velocity diverged from its experimentally measured ones beyond a wind speed of 10 m s^−1^, we decided to back calculate values of *S* from Krall's experimental data for the nitrous oxide–water system. Equation (3.12) can be written as

4.1 $$k_{L}=\alpha \sqrt{DS},$$

where

4.2 $$\alpha =\frac{\Gamma (a+1/2)}{\Gamma (a+1)}\sqrt{\frac{2a+1}{\pi }}.$$

For *a* = 0 (i.e. the conventional Danckwerts age distribution function, equation (4.2) gives *α* = 1, whereas for a→∞ (i.e. high wind speed), it can be shown that $\alpha \to \sqrt{2/\pi }≅0.797885$ (see also table 1 in the appendix (electronic supplementary material, S1) of the manuscript of Mondal & Chatterjee [29]) and the surface age distribution function $f(t,t_{p}\to \infty )\to S\delta (St-0.5)$ where *δ*(*t*) is the delta function. Thus, the value of *α* is bounded within these limits and an average value can be used for all wind speeds, i.e.

4.3 $$\alpha ≅0.5+\sqrt{\frac{1}{2\pi }}\approx 0.9.$$

**Figure 5.**
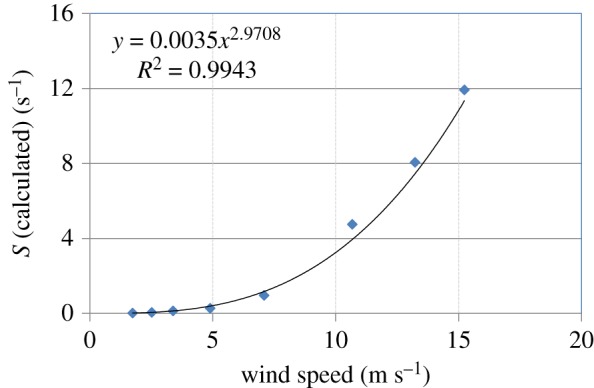
Calculated values of the fundamental surface renewal rate *S* from equation (4.1) as a function of wind speed (at 10 m) for the nitrous oxide–water system at 20°C. Values of *α* = 0.9, *D* = 1.88 × 10^−9^ m^2^ s^−1^ and the transfer-velocity data shown in fig. 7.11 in the work of Krall [23] were used in the calculations.

Thus, $k_{L}\approx 0.9\sqrt{DS}$ which can be compared to $k_{L}=\sqrt{DS}$, which is the expression for the transfer velocity for the conventional Danckwerts model (i.e. *a* = 0). The uncertainty in measured values of *k*_L_, which can sometimes be substantial, provides a justification for using the approximation *α* = 0.9 in equation (4.1). Figure 5 shows values of *S* calculated from all the experimental values of *k*_L_ for the nitrous oxide–water system (at 20°C and for a clean interface) reported in fig. 7.11 in Krall's thesis as a function of wind speed (at 10 m) using equation (4.1) with *α* = 0.9 and *D* = 1.88 × 10^−9^ m^2^ s^−1^. It is clearly seen that the fundamental frequency quantum *S* can be represented very well with the power-law equation shown in figure 5. As the wind speed increases by a factor of 9, *S* increases by a factor of 553, which shows the dramatic influence of the wind speed on the basic surface renewal rate or frequency quantum.

As was discussed earlier and as can be also observed from equation (3.12), the surface renewal model predicts that $k_{L}\propto D^{0.5}$, i.e. the Schmidt number exponent (−*m*) equals −1/2. Although there is some uncertainty, the data for the trace gases used by Krall [23] showed the exponent gradually changing from −2/3 (smooth surface) to −1/2 (wavy surface) as the wind speed increased. (It has been reported by Jähne [36] that measured values of *m* are never larger than 0.75 for air–water heat and gas transfer (see fig. 4.8 on page 110 in his work).) For example, Krall's fig. 7.14 shows that, for the clean air–water interface, *m* changed from 0.66 to 0.52 as the wind speed changed from 2.4 to 10.6 m s^−1^, with the midpoint being at around a wind speed of 4.5 m s^−1^. This behaviour can be attributed to a preponderance of short-lived liquid elements (i.e. those that are not completely penetrated by the dissolved gas) at the air–water interface at higher wind speeds when *S* will be high due to increased turbulence. The change in the value of *m* was observed even when there was a surfactant (triton) present on the water surface in trace amounts. For a surfactant dosage of 0.26 µmol l^−1^ of triton, the water surface was smooth until a wind speed of 7 m s^−1^ and became wavy at wind speeds greater than 11 m s^−1^, with the midpoint of the transition being at a wind speed of 9 m s^−1^. The surface renewal model presented in this work is unable to capture this behaviour of a changing *m*, and thus offers only a partial glimpse into reality, which is a characteristic feature of scientific models [37]. The semi-empirical facet model, which arbitrarily apportions the total water surface into two parts, one wavy and the other smooth, can capture this behaviour [22,23]. Another possibility of modelling this feature is to arbitrarily assume that the basic or fundamental surface renewal rate *S* or renewal time is itself depth-dependent [23,38]. However, this would entail abandoning the concept of *S* being a fundamental frequency quantum—a characteristic feature of the level of turbulence or flow instability, which was the underlying theoretical premise of the prior work of Mondal & Chatterjee [29], and is thus also the basis of this work. In our opinion, a more fundamental and theoretically elegant approach would be to retain the concept of the basic frequency quantum *S* but combine it with the film-penetration concept discussed earlier, while using the GD framework in the mathematical description. Such a mass-transfer model, whose development is beyond the scope of the current manuscript, may be able to provide a theoretical explanation for the empirical observation of the transitioning of the Schmidt number exponent with an increase of wind speed, which has been observed in air–water gas exchange.

## 5. Concluding remarks

This work extended the steady-state two-parameter generalized Danckwerts age distribution, presented by Mondal and Chatterjee [29], to unsteady-state conditions. For the three different wind speeds used by Garbe *et al.* [30] in their experiments on air–water heat exchange on the Heidelberg Aeolotron, calculations showed that the age distribution has a sharp peak initially, and thereafter flattens out and acquires a bell-shaped character with the progress of process time. The time taken by the distribution to attain a steady-state shape is strongly but inversely correlated with wind speed. It takes approximately 150, 9 and 4 s to reach the steady state at wind speeds of 2, 4.2 and 8 m s^−1^. Also, with an increase of wind speed (i.e. greater turbulence at the gas–liquid interface), there is a narrowing of the age distribution, with decreased skewness and a larger peak. The mean eddy renewal or burst time increases linearly with process time in the initial period and asymptotically attains a final steady-state value, which is approximately 20.83, 2.86 and 1.33 s for the wind speeds of 2, 4.2 and 8 m s^−1^, respectively. Thus, the average eddy burst time decreases dramatically with an increase in wind speed showing the great effect of turbulence. The GD distribution was used in the analysis of transient absorption of a gas into a large body of liquid, assuming negligible gas-side mass-transfer resistance. Explicit mathematical expressions were derived for the liquid-side mass-transfer coefficients for absorption of the gas at the gas–liquid interface and its subsequent transfer to the bulk liquid under unsteady-state conditions. These expressions were used to calculate the gas-absorption and dissolved-gas transfer coefficients for the absorption of oxygen in water at 25°C at the three different wind speeds used by Garbe *et al.* [30]. Under unsteady-state conditions, these two coefficients are not equal and have an inverse relationship, which indicates a heightened accumulation of dissolved gas in the liquid elements at the surface of a turbulent liquid, especially during the initial moments of absorption. Depending upon the turbulence level, this phenomenon is expected to last from a few seconds to a few minutes. With the progress of absorption towards steady state, however, both coefficients approach each other. Finally, theoretical predictions of the steady-state mass transfer coefficient or transfer velocity were in fair agreement (average absolute error of prediction = 18.1%) with some experimental measurements of Krall [23] of the same for the nitrous oxide–water system at 20°C that were made on the Heidelberg Aeolotron.

## Supplementary Material

SageMath code for modelling unsteady-state physical gas absorption in a large volume of liquid using the generalized Danckwerts age distribution function

## Data accessibility

All theoretical calculations presented herein were performed with the equations developed in this paper. A sample SageMath code used in performing the calculations is provided in the electronic supplementary material. The experimental data referred to in this manuscript can be found in the prior works of Richter & Jähne [22], Krall [23], Garbe *et al*. [30] and Hutchinson & Sherwood [33].
