# Supplementary material for: A surface renewal model for unsteady-state mass transfer using the generalized Danckwerts age distribution function
Source: R Soc Open Sci. 2018 May 23;5(5):172423. doi: 10.1098/rsos.172423 (PMC5990727; doi:10.1098/rsos.172423)
Supplement: SageMath code for modelling unsteady-state physical gas absorption in a large volume of liquid using the generalized Danckwerts age distribution function [file rsos172423supp1.pdf]

# Sample SageMath code for modelling the unsteady-state physical absorption of a gas into a large volume of liquid using the generalized Danckwerts (GD) age distribution function. This code was used for performing calculations presented in the manuscript entitled "A surface renewal model for unsteady-state mass transfer using the generalized Danckwerts age distribution function" (authors: Isabelle. R. Horvath and Siddharth. G. Chatterjee), which has been accepted by Royal Society Open Science.

```
# Unsteady state physical gas absorption in a large volume of liquid using the generalized Danckwerts (GD) age distribution function

var('x y')

# Input parameters
u = 2 # Wind speed in the experiments of Garbe et al. (2002) in m/s
D = 2.12*10^(-9) # Diffusion coefficient of oxygen in water at 25 C in m^2/s
a = 0.5 # Parameter of GD distribution
S = 0.036 # Frequency quantum in 1/s
# End of input parameter list

# Unsteady-state GD age distribution function

f_GD(x,y) = S*(2*a + 1)^(a + 1)*(S*x)^a*exp(-(2*a + 1)*S*x)/(gamma(a + 1) - gamma_inc(a + 1, (2*a + 1)*S*y)) # GD age distribution [Equation (2.6)]

# Create an empty plot object

B = plot([],figsize=(5,5),axes_labels=['age t (s)','age distribution f(t, tp) (1/s)'],gridlines=True, frame=true, xmax = 100, ymax = 0.12)
B += plot(f_GD(x,10),x,[0,10],color='purple', legend_label='tp = 10 s', linestyle="dotted", thickness=1.5)
B += plot(f_GD(x,15),x,[0,15],color='red', legend_label='tp = 15 s', linestyle="dashed", thickness=1.5)
B += plot(f_GD(x,40),x,[0,40],color='black', legend_label='tp = 40 s', linestyle="dashdot", thickness=1.5)
B += plot(f_GD(x,100),x,[0,100],color='blue', legend_label='tp = 100 s', linestyle="solid", thickness=1.5)
B += text("wind speed = 2 m/s", (0.72,0.4), alpha=0.8, fontsize='large', fontweight='regular', color='black', axis_coords='True')
show(B)

# Mass transfer coefficients for gas absorption at the gas-liquid interface and dissolved-gas transfer to the bulk liquid

kLabs(y) = sqrt(D*S*(2*a + 1)/n(pi))*(gamma(a + 1/2) - gamma_inc(a + 1/2, (2*a + 1)*S*y))/(gamma(a + 1) - gamma_inc(a + 1, (2*a + 1)*S*y)) # Equation (3.6)
```

```
kLtrans(y) = sqrt(D*4*S/(n(pi)*(2*a + 1)))*(gamma(a + 3/2) - gamma_inc(a + 3/2, (2*a + 1)*S*y))/(gamma(a + 1) - gamma_inc(a + 1, (2*a + 1)*S*y)) # Equation (3.10)

# Create an empty plot object

B = plot([],figsize=(5,5),axes_labels=['process time tp (s)','mass transfer coefficient kL (m/s)'],gridlines=True, frame=true, xmax = 100, ymax = 6*10^(-5))
B += plot(kLabs(y),y,[0,100],color='black', legend_label='absorption', thickness=1.5)
B += plot(kLtrans(y),y,[0,100],color='red', legend_label='transfer', linestyle='dashed', thickness=1.5)
B += text("wind speed = 2 m/s", (0.72,0.4), alpha=0.8, fontsize='large', fontweight='regular', color='black', axis_coords='True')
show(B)

# Mean eddy renewal time

tren(y) = (1/S/(2*a + 1))*(gamma(a + 2) - gamma_inc(a + 2, (2*a + 1)*S*y))/(gamma(a + 1) - gamma_inc(a + 1, (2*a + 1)*S*y))
B = plot([],figsize=(5,5),axes_labels=['process time tp (s)','mean eddy renewal time tren (s)'],gridlines=True, frame=true, xmax = 100)
B += plot(tren(y),y,[0,100], thickness=1.5)
B += text("wind speed = 2 m/s", (0.72,0.4), alpha=0.8, fontsize='large', fontweight='regular', color='black', axis_coords='True')
show(B)

# Sample calculations

trenss = (a + 1)/S/(2*a + 1)
print "Steady state mean eddy renewal time = ", trenss, " s"
print "kLabs (tp = 150 s) = ", kLabs(150), " m/s"
print "kLtrans (tp = 150 s) = ", kLtrans (150), " m/s"
kL_steadystate = sqrt(D*S*(2*a + 1)/n(pi))*gamma(a + 1/2)/gamma(a + 1) # Equation (3.12)
print "kL_steadystate = ", kL_steadystate
```
